# How useful can the Voigt profile be in protein folding processes?

**DOI:** 10.1007/s10930-020-09954-5

**Published:** 2021-01-05

**Authors:** Luka Maisuradze, Gia G. Maisuradze

**Affiliations:** 1 Present address: Department of Molecular Biophysics and Biochemistry, Yale University, New Haven, CT, 06520-8114, USA; 2 Baker Laboratory of Chemistry and Chemical Biology, Cornell University, Ithaca, NY 14853-1301, USA

**Keywords:** Voigt profile, Levy flight, Gaussian and Lorentzian functions, protein folding, molecular dynamics simulations, quasielastic neutron-scattering spectra

## Abstract

The analytical expression for the Voigt profile, along with its simplified forms for the Gaussian and Lorentzian dominance, is presented. The applicability of the Voigt profile in the description of anomalous diffusion phenomena, ubiquitous in different fields of science including protein folding, is discussed. It is shown that the Voigt profile is a good descriptor of the processes occurring in protein folding and in the native state. The usefulness of the Voigt profile in deriving important information of the diffusive motions in proteins from a quasielastic incoherent neutron scattering experiments is illustrated.

Many mathematical functions are successfully used in describing ubiquitous physical phenomena across multiple disciplines of natural science. One such function is the Voigt profile [1], a widely-used function in astrophysical [2–4], Raman [5,6], plasma [7] and applied [8] spectroscopy, which is a convolution of Gaussian, $G(x)=\left(2/\sqrt{\pi }\omega_{G}^{\prime }\right)\text{exp}\left[-{\left(x/\omega_{G}^{\prime }\right)}^{2}\right]$, and Lorentzian, $L(x)=(1/\pi )\left[\omega_{L}/\left(x^{2}+\omega_{L}^{2}\right)\right]$, functions, and can be expressed in different forms [2–9]:

(1a) $$V(v,\xi )=\left(\xi /\pi^{3/2}\right)\overset{\infty }{\underset{-\infty }{\int }}\frac{\text{exp}\left(-y^{2}\right)}{\xi^{2}+{\left(v-y\right)}^{2}}dy$$

(1b) $$V(v,\xi )=(1/\pi )\overset{\infty }{\underset{0}{\int }}\text{exp}\left(-\xi y-y^{2}/4\right)\text{cos}(v\;y)dy$$

(1c) $$V(v,\xi )=(1/\pi \xi )\overset{\infty }{\underset{0}{\int }}\text{exp}\left[-y-{\left(y/2\xi \right)}^{2}\right]\text{cos}(vy/\xi )dy$$

where $\omega_{G}\left[\omega_{G}=\sqrt{\text{ln}2}\omega_{G}^{\prime }\right]$ and *ω*_*L*_ are Gaussian and Lorentzian half-widths at half-maximum (HWHM), respectively; $\xi =\omega_{L}/\omega_{G}^{\prime }$ is the line-damping parameter and $v=x/\omega_{G}^{\prime }$. It can easily be shown from Eq. (1) that for limiting cases of *ξ*, the Voigt profile obtains Gaussian (*ξ* → 0) and Lorentzian (*ξ* → ∞) form. The Voigt profiles for different *ξ* along with Gaussian and Lorentzian functions are illustrated in Fig. 1. The dynamics of the change from the Gaussian to Lorentzian function is clear on both panels. Unlike the Gaussian and Lorentzian functions, the Voigt profile cannot be expressed in compact analytical form. For many years, tables [10], computer algorithms [11], and analytical interpretation formulae with limited ranges of *ξ* and *υ* [12] were used for calculation of the Voigt profile. Combining *ξ* and *υ* into the complex variable *z* = *υ* + *iξ*, the Voigt profile can be represented as the real part of the complex function [13]

(2) $$V(v,\xi )=\text{Re}[W(z)],\;W(z)=\frac{i}{\pi }\int_{-\infty }^{\infty }\frac{e^{-y^{2}}}{z-y}dy$$

Also, the function *W(z)* is closely related to the complex complementary error function *erfc(-iz)* [14] and the Dawson function $F(z)=\int_{0}^{z}e^{y^{2}}dy$

(3) $$W(z)=e^{-z^{2}}\text{erfc}(-iz)=e^{-z^{2}}\left\{1+\frac{2i}{\sqrt{\pi }}\int_{0}^{z}e^{y^{2}}dy\right\}=e^{-z^{2}}+\frac{2i}{\sqrt{\pi }}F(z)$$

Both error and Dawson functions were approximated by Chebyshev polynomials [16–18].

We have derived an analytical form for *V*(*υ*, *ξ*) which is both exact for arbitrary values of *ξ* and *υ* and sufficiently simple to be very useful in the numerical extraction of lineshape parameters from spectral data [5,6,9]. It has the following form:

(4) $$V(v,\xi )=\frac{\text{exp}\left(-v^{2}\right)}{\sqrt{\pi }}\left\{\text{exp}\left(\xi^{2}\right)\text{cos}(2\xi v)[1-\text{coth}(2\pi \xi )]+\frac{2\xi }{\pi }\overset{\infty }{\underset{n=-\infty }{\sum }}\frac{\text{exp}\left(-\frac{1}{4}n^{2}\right)\text{cosh}(nv)}{n^{2}+4\xi^{2}}\right\}$$

with a rapidly converging and easily tractable series. The Gaussian and Lorentzian components in Eq. (4) are predominantly represented by the first term and by the sum, respectively. Therefore, Eq. (4) can be reduced to simpler limiting forms of Gaussian and Lorentzian dominance, respectively [9]:

(5a) $$V(v,\xi )\approx (1/\sqrt{\pi })\text{exp}\left(-v^{2}\right)\{\text{exp}\left(\xi^{2}\right)\text{erfc}(\xi )\text{cos}(2\xi v)+\;\left[2{\text{sin}}^{2}(\xi v)/\sqrt{\pi }\xi \right]\left[1+0.1379v^{2}+\left(0.0120+0.0434\xi^{2}\right)v^{4}\right]\},\;v\ll 1$$

(5b) $$V(v,\xi )\approx \text{exp}\left(-v^{2}\right)[a(\xi )+b(\xi )\text{cos}(2\xi v)],\;a={\left(\pi \xi \right)}^{-1};b=\pi^{-1/2}\left[\text{exp}\left(\xi^{2}\right)\text{erfc}(\xi )-{\left(\sqrt{\pi }\xi \right)}^{-1}\right],\;v\ll 1,\xi \ll 1$$

(6a) $$V(v,\xi )\approx \left(2\xi /\pi^{3/2}\right)\overset{\infty }{\underset{n=-\infty }{\sum }}\text{exp}\left[-{\left(v-n/2\right)}^{2}\right]/\left(n^{2}+4\xi^{2}\right),\;\xi \geq 1$$

(6b) $$V(v,\xi )\approx \frac{1}{\pi }\frac{\xi }{\xi^{2}+v^{2}}\left[1+\frac{v^{2}}{{\left(\xi^{2}+v^{2}\right)}^{2}}\right],\;\xi \gg 1$$

These equations are valid close to the maximum (Eq. 5), intermediate range (Eq. 6a) and far from the maximum (Eq. 6b).

It should be noted that the analytical form with a rapidly converging and easily tractable series of the imaginary part of the complex function *W(z)* was also derived in Refs. [5,6].

The property of covering the whole spectrum of band shape variations from the Gaussian to the Lorentzian function makes the Voigt profile an applicable function to the description of a number of physical, biological and social phenomena, including protein folding processes. The point is that the Levy flights [19], the presence of which usually leads to anomalous diffusion [20,21], an observed phenomenon in protein folding [22–25], intermittent chaotic systems [26], bacterial motion [27], and foraging biology [28], have infinite variance (except the Gaussian distribution) and an analytical form is known only for a few special cases. For example, the symmetrical Levy stable distribution of index α (0 <*α* ≤ 2) and scale factor γ (γ > 0), which has the following form [29]

(7) $$P_{\text{Levy}}(v)=\frac{1}{\pi }\overset{\infty }{\underset{0}{\int }}\text{exp}\left(-\gamma y^{\alpha }\right)\text{cos}(vy)dy$$

can be reduced to the Gaussian (α = 2) and Lorentzian (α = 1) distributions as its special cases. These are only two symmetrical Levy distributions that can be expressed as elementary functions. It is necessary, therefore, to find analytical functions which can cover the region 1 < α < 2. To this end, the Voigt profile might be a useful function.

In order to resolve the second discouraging mathematical property of Levy flights, the lack of finite variance, Mantegna and Stanley introduced the truncated Levy flight (TLF), in which the arbitrarily large steps of the Levy flight are eliminated [29]. A TLF is characterized by probability distribution

(8) $$T(v)\equiv \{\begin{array}{l} 0,\\ cP_{\text{Levy}}(v),\;\\ 0 \end{array}\;\begin{array}{l} v<-l\\ -l\leq v\leq l\\ v>l \end{array}$$

where *c* and *l* are the normalizing constant and cutoff length, respectively. It is worth noting that the conditions, imposed on *υ* in Eq. (8) to make the variance finite, are embedded in the Voigt profile (1c). The reciprocal line-damping parameter 1/*ξ*, which varies in the range (0, ∞) depending which distribution is dominant, can control the cutoff length. If the distribution is close to Gaussian then 1/*ξ* → ∞, and when the distribution is close to Lorentzian then 1/*ξ* → 0. In a Gaussian distribution, the range of *υ/ξ* can be (− ∞,∞) since Gaussian distribution has finite variance without any conditions. However, with increment of Lorentzian dominance 1/*ξ* gets smaller and the range of *υ/ξ* decreases, which is consistent with the idea of TLF.

Pagnini and Mainardi [30] proposed a probabilistic generalization of the Voigt profile as the convolution of two arbitrary symmetric Levy distributions. They introduced parametric integro-differential equations, classified as space-fractional diffusion equations of double order, for both the ordinary and the generalized Voigt profiles. Moreover, the Voigt profile was expressed in terms of the Mellin-Barnes integrals, Fox H-function and Meijer G-function [31]. All three functions were introduced into physics by Schneider [32] as analytic representations for Levy distributions, and as solutions of fractional equations [21]. Plus, Fox H-functions enable to treat several phenomena including anomalous diffusion in a unified and elegant framework [33].

Based on these studies, it is of interest to investigate whether the Voigt profile can be useful for describing different important biological processes. In this Letter, along with some interesting aspects of the Voigt profile, we discuss its applicability to one of the most important biological processes - protein folding [22–25, 34]. In particular, we examine the dependence of the variance of the Voigt profile on the line-damping parameter *ξ*, and treat probability distribution functions (PDFs) of some global and local coordinates of protein folding trajectories with the Voigt profile. However, before presenting the results, we briefly describe the methodology used in the presented study. The data analyzed in this work were obtained from molecular dynamics (MD) trajectories generated with the coarse-grained united-residue (UNRES) force field [35, 36] and the all-atom optimized potentials for liquid simulations (OPLS) force field [37]. In particular, we carried out (i) forty-eight coarse-grained MD simulations of one of the mutants, L26D (PDB ID: 2N4R) [38], of the Formin binding protein 28 (FBP28) WW domain (PDB ID: 1E0L) [39] at two different temperatures (305K, 315K) (24 MD trajectories, with ~1.4 μs UNRES time, at each temperature); and (ii) five all-atom MD simulations of α/β model protein VA3 (PDB ID: 1ED0), each of a duration of 80 ns, at 300 K in explicit water [40]. All coarse-grained MD simulations start with the same initial fully-extended structure of L26D but with different velocities, whereas all-atom MD simulations start with the same initial structure of VA3, taken from the NMR model 1 [41], but with different velocities.

Because of the obvious dependence of the variance of the Voigt profile on the line-damping parameter *ξ*, we have studied a variance of the Voigt profile, which has the following form:

(9) $$Var(\xi )=\overset{\infty }{\underset{-\infty }{\int }}v^{2}V(v,\xi )dv=\frac{1}{\sqrt{\pi }}\text{exp}\left(\xi^{2}\right)[1-\text{coth}(2\pi \xi )]\overset{\infty }{\underset{-\infty }{\int }}v^{2}\text{exp}\left(-v^{2}\right)\text{cos}(2\xi v)dv+\frac{2\xi }{\pi^{3/2}}\overset{\infty }{\underset{-\infty }{\int }}v^{2}\text{exp}\left(-v^{2}\right)\overset{\infty }{\underset{n=-\infty }{\sum }}\frac{\text{exp}\left(-0.25n^{2}\right)\text{cosh}(nv)}{n^{2}+4\xi^{2}}dv$$

By integrating (9) we obtain

(10) $$Var(\xi )=\frac{1}{2}[1-\text{coth}(2\pi \xi )]\left(1-2\xi^{2}\right)+\frac{\xi }{\pi }\overset{\infty }{\underset{n=0}{\sum }}\frac{n^{2}+2}{n^{2}+4\xi^{2}}$$

where the series on the right-hand side is divergent, consequently the variance of the Voigt profile is undefined. However, since the Voigt profile can be presented in simpler forms for Gaussian and Lorentzian dominance, it is worthwhile to study the variance for these approximations.

It is clear from the Voigt profiles with Lorentzian dominance (Eq. (6a) contains the sum, the variance of which is a divergent series, and the first term of Eq. (6b) has Lorentzian function form) that the variance of these approximations is undefined. Using the integrals of elementary functions [42], we have obtained variance of the Voigt profiles of Gaussian dominance, Eqs. (5a) and (5b), respectively:

(11a) $$Var(\xi )=(1/2)\left(1-2\xi^{2}\right)\text{erfc}(\xi )+\frac{1-\text{exp}\left(-\xi^{2}\right)}{\sqrt{\pi }\xi }\left(0.6259+0.0814\xi^{2}\right)+\left(\text{exp}\left(-\xi^{2}\right)/\sqrt{\pi }\right)\left[1.5486\xi +0.2605\xi^{3}-0.3136\xi^{5}+0.0434\xi^{7}\right]$$

(11b) $$Var(\xi )=(1/2)\left(1-2\xi^{2}\right)\text{erfc}(\xi )+\frac{1}{2\sqrt{\pi }\xi }\left[1-\left(1-2\xi^{2}\right)\text{exp}\left(-\xi^{2}\right)\right]$$

Figure 2 shows the variance as a function of *ξ* calculated from Eqs. (11a) and (11b). Since Eq. (5b) is a good approximation of the Voigt profile for *ξ* << 1, the variance of this approximation is correct only for small *ξ*, and it coincides with the variance of the more general approximation, Eq. (5a), in the region of small *ξ*. The persistence of variance for small *ξ* indicates that the Voigt profile is almost purely Gaussian. There are two reasons for the small increase and then decrease of variance (solid line in Fig. 2) in the region 0.01 < *ξ* < 3.0. First, Eq. (5a) is an approximation of Eq. (4), and it is accurate in the region of small *υ*[9]. Second, the region 0.01 < *ξ* < 3.0 is a transition region from the Gaussian to the Lorentzian function, consequently variance cannot be a constant value. For *ξ* > 3 variance increases, because the Voigt profile is becoming a Lorentzian function, and when *ξ* → ∞ variance will be undefined. The reason of relatively slow increase is the approximation mentioned above. The decrease of variance (dash line), defined by Eq. (11b), in the region of large *ξ* is caused by the incorrectness of Eq. (5b) in this region of *ξ*.

Starting from the famous experiments of Anfinsen et al. [34], the question of how proteins reach their biologically active ensembles of conformations still remains to be answered. The selection of a correct model for protein folding kinetics and the coordinates along which the intrinsic folding pathways can be identified in order to interpret experimental data still remains challenging. The common choices for reaction coordinates are root-mean-square-deviation (RMSD) with respect to the native structure, radius of gyration (Rg), number of native contacts, and other order parameters. Here, we examine the PDFs of radius of gyration of folding trajectories of L26D mutant of the FBP28 WW domain, generated with the coarse-grained UNRES force field, by the Voigt profile. There are ten and eleven folding trajectories at 305K and 315K, respectively. Depending on how fast the protein folds, the PDF of R_g_ can be either unimodal or bimodal (Fig. 3A–B). By fitting PDFs of R_g_ (the modes corresponding to native states in bimodal PDFs) of the folding trajectories, we found that the faster a protein folds, i.e. the longer it remains in the native state, the smaller the value of the line-damping parameter *ξ* becomes, indicating the increase of Gaussian dominance in the PDF of Rg (Fig. 3C). By fitting PDFs of Rg for the modes corresponding to unfolded states in bimodal PDFs, we found the same behavior for the line-damping parameter *ξ*, i.e. the longer the proteins stays in the unfolded state, the smaller the value of the line-damping parameter *ξ* becomes (Fig. 3D). Moreover, the Lorentzian contribution in both cases increases with the temperature (Fig. 3C–D). These findings can be explained as follows: the first, that the PDFs of R_g_ of protein can be described by the Voigt profile, is not surprising given that the PDF of R_g_ of a flexible polymer may be written in terms of the Chebyshev polynomial [43] which, as was mentioned above, itself is related to the Voigt profile [16–18]; the second, in fast-folding trajectories, the system spends a short time in the unfolded state, and makes long jumps to proceed over the transition-state barrier to the native state, which consequently increases the Lorentzian contribution in the PDFs of R_g_ of the unfolded state. With the increase of temperature the number of long jumps increases, which is reflected in the shape of PDFs of Rg by the increase of the Lorentzian contribution.

In the next example, we successfully apply the Voigt profile to PDFs of local coordinates. In particular, in our recent study [40] on the example of the α/β model protein VA3, we investigated the rotational correlation functions of the backbone N-H bonds and of the dihedral angles *γ* in order to understand how the main chain in the native state of a protein fluctuates on different time scales. The orientation of the backbone of a protein around a residue *n* at any time *t* can be characterized by a unit vector *un(t)* representing the orientation of a local probe of the protein dynamics in a frame attached to the molecule. One of the probes, *un(t)*, of the backbone dynamics, considered in our previous study [40], represents the orientation of the main chain measured by a coarse-grained dihedral angle *γ*_*n*_ built on four consecutive *C*^α^ atoms [44]. The probability that the vector ***u*** (see Fig. S1 in ref. [40]) is rotated by an angle Δ*γ* after a time *t* > 0 is represented by the quantity *F*(Δ*γ*, *t*)*dγ* For a free-diffusion equation on a circle with diffusion coefficient *D*(*t*), we found that the PDF *F*(Δ*γ*, *t*) is a series of Chebyshev polynomials (see Eq. S17 in ref. [40]). The analytical solutions *F*(Δ*γ*, *t*) agreed quite well with the PDFs computed by MD for different residues (see Fig. S8 (a-c) in ref. [40]); however, there are some discrepancies in the PDFs for the *γ* angles with multiple-minima free-energy profiles (FEPs) (see Fig. S8 (b, c) in ref. [40]). Because of the correlation between the Chebyshev polynomials and Voigt profiles, here, we apply the Voigt profile to the PDF *F*(Δ*γ*, *t*) illustrated in Fig. S8 of ref. [40]. Figure 4 shows a perfect fit of the Voigt profile (green line) with the PDFs computed by MD (black line) for all three *γ* angles with the line-damping parameters: *ξ* = 0.011 (A), 0.661 (B), 0.833 (C). As was expected, the line-damping parameters, *ξ*, for PDF *F*(Δ*γ*, *t*) of the *γ* angle with a typical harmonic FEP illustrates a strong Gaussian dominance (panel A), whereas in the other two *γ* angles with multiple-minima FEPs *ξ* becomes greater indicating an increase of the Lorentzian contribution (panels B, C). In Fig. 4, for the comparison, we keep curves calculated by Eqs. S17 (red line) and S30 (blue line) of ref. [40].

Another field in which the Voigt profile can be successfully applied is quasielastic incoherent neutron scattering [45], one of the commonly-used experimental techniques to understand the molecular motion involved in protein folding. In the quasielastic incoherent approximation, the theoretical scattering function describing the internal motion in the protein can be expressed by [46,47]

(12) $$S_{\text{theor}}(Q,\omega )=\text{exp}\left(-Q^{2}\langle u^{2}\rangle /3\left[A_{0}(Q)\delta (\omega )+\overset{n}{\underset{i=1}{\sum }}A_{i}(Q)L\left(\omega ,\Gamma_{i}\right)\right]\right)$$

where *Q* is the neutron momentum transfer, < *u*^2^ > is the mean square amplitude of vibrations, *A0*(*Q*)δ(ω) is the elastic term with an infinitely high spectrometer energy resolution δ(ω), and the quasielastic component *Ai*(*Q*)*L*(ω,Γ*i*), which measures the mobility of the protons within protein, is the sum of Lorentzian functions. However, the experimentally measured scattering function is that of Eq. (12) convoluted with the instrumental (spectrometer) Gaussian type resolution function, consequently the overall quasielastic incoherent scattering function is a convolution of these two functions, i.e. the Voigt profile. In order to correctly define the diffusive motions in proteins, the proper determination of the Gaussian and Lorentzian contributions in experimentally measured scattering function is required. This can easily be achieved by fitting the experimental data with the Voigt profile [5,6].

As an example, we fitted quasielastic neutron-scattering spectra of lysozyme in deuterated glycerol for different temperatures (300K, 330K, 370K and 400K) [48] by the Voigt profile, and obtained the line-damping parameter ξ as a function of temperature. It turns out that, if we fit the entire quasielastic neutron-scattering spectra (see Figs. 3 and 4 in Ref. [48]), the line-damping parameter ξ increases with the increase of temperature (Fig. 5), which indicates an increment of Lorentzian contribution. In other words, the line-damping parameter ξ is a good descriptor of the substantial rising of the quasielastic intensity due to the increase of temperature. If we fit quasielastic neutron-scattering spectra with the Voigt profile focusing on a perfect fit of only the high energy region, then the obtained value of the line-damping parameter ξ is very small, indicating Gaussian dominance in the Voigt profile. This finding is in harmony with the previous study [48], in which the authors fitted the same spectra by the Gaussian and Lorentzian functions, and found that the Gaussian function gives quite a good fit in the high energy region ( > 1 meV), whereas the Lorentzian function fits the region near the elastic peak better (see Fig. 4 in Ref. [48]).

Finally, as it was mentioned above, the Voigt profile is related to the probability integral, and it is a real part of the complex function *W*(*υ*, *ξ*) [Eq. (2)]. Studying experimental results of resonance Raman and absorption spectra [5,6], we found that when the broadening parameters are not small, inclusion of the imaginary part of *W*(*υ*, *ξ*), the analytical forms of which are available in Refs. [5,6], in the expression along with the Voigt profile is important [5,6].

In our opinion, the ideas discussed in this work might be important for different fields of science. It is inevitable that in most physical systems the power-law tail of the Levy flight is truncated at a characteristic scale that often is the system size [49]. For example, most biological systems are bounded/limited (cell trajectories are limited by the cell cycle and environmental conditions), resulting in the truncation of the power law tail, which introduces a characteristic scale to the movement pattern [50]. Therefore, the Voigt profile, which inherently includes the conditions imposed on Levy flight for truncation, might be a useful function for investigating these processes. Moreover, we illustrated that the Voigt profile can be a good descriptor of the processes occurring in protein folding and in native state. Therefore, applications of the Voigt profile on different biological processes are planned in the nearest future.

## Figures and Tables

**Fig. 1:**
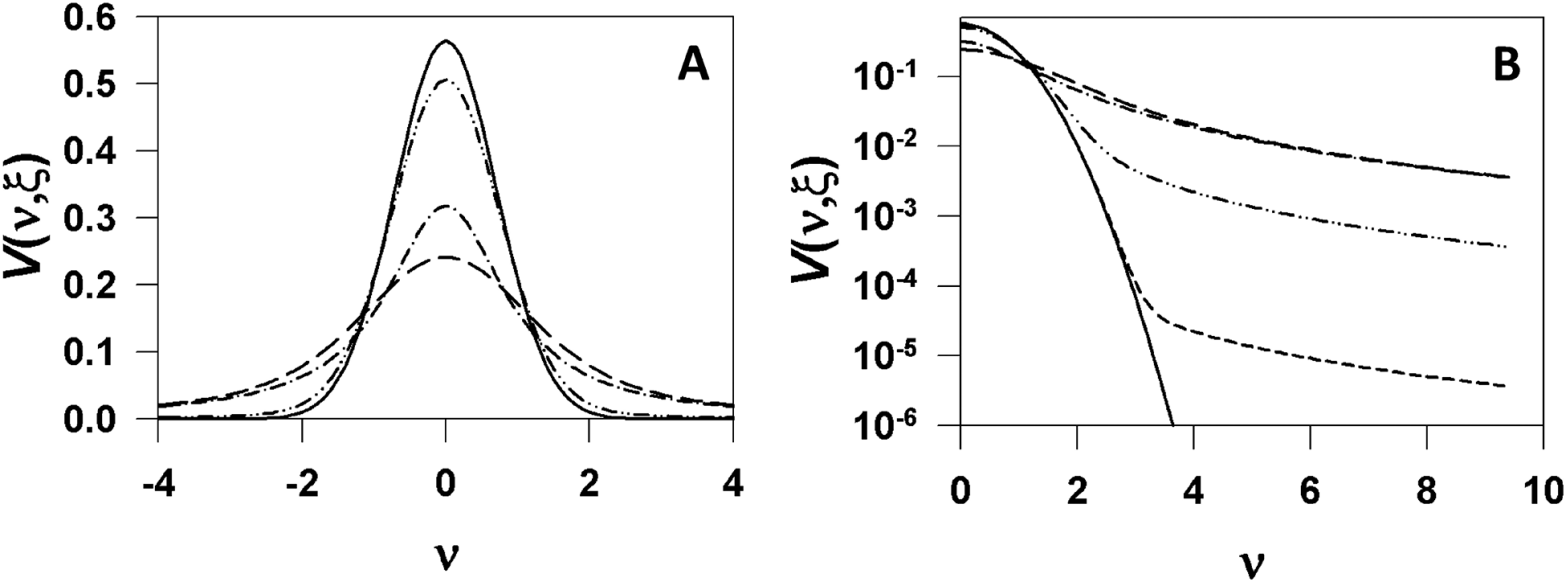
(A) Gaussian (solid line) and Lorentzian (dash-dot line) functions along with the Voigt profiles for different line-damping parameters ξ [dash-dot-dot line (ξ = 0.1), long-dash line (ξ = 1.0)]; (B) The same functions as in (A) plus the Voigt profile for ξ = 0.001 (short-dash line) only ordinate is in log scale

**Fig. 2:**
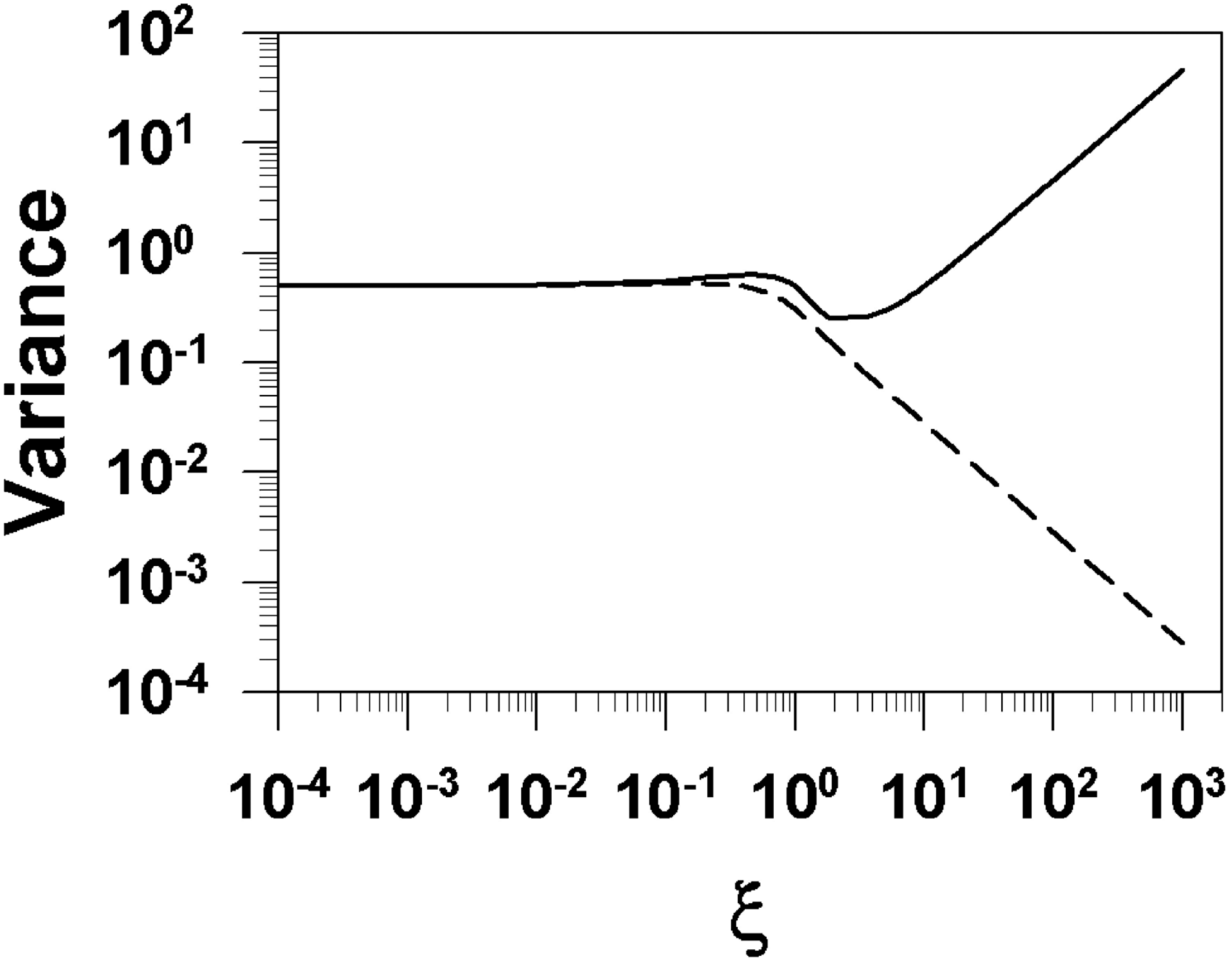
Variance as a function of ξ calculated by Eq. (11a) (solid line), and Eq. (11b) (dash line)

**Fig. 3:**
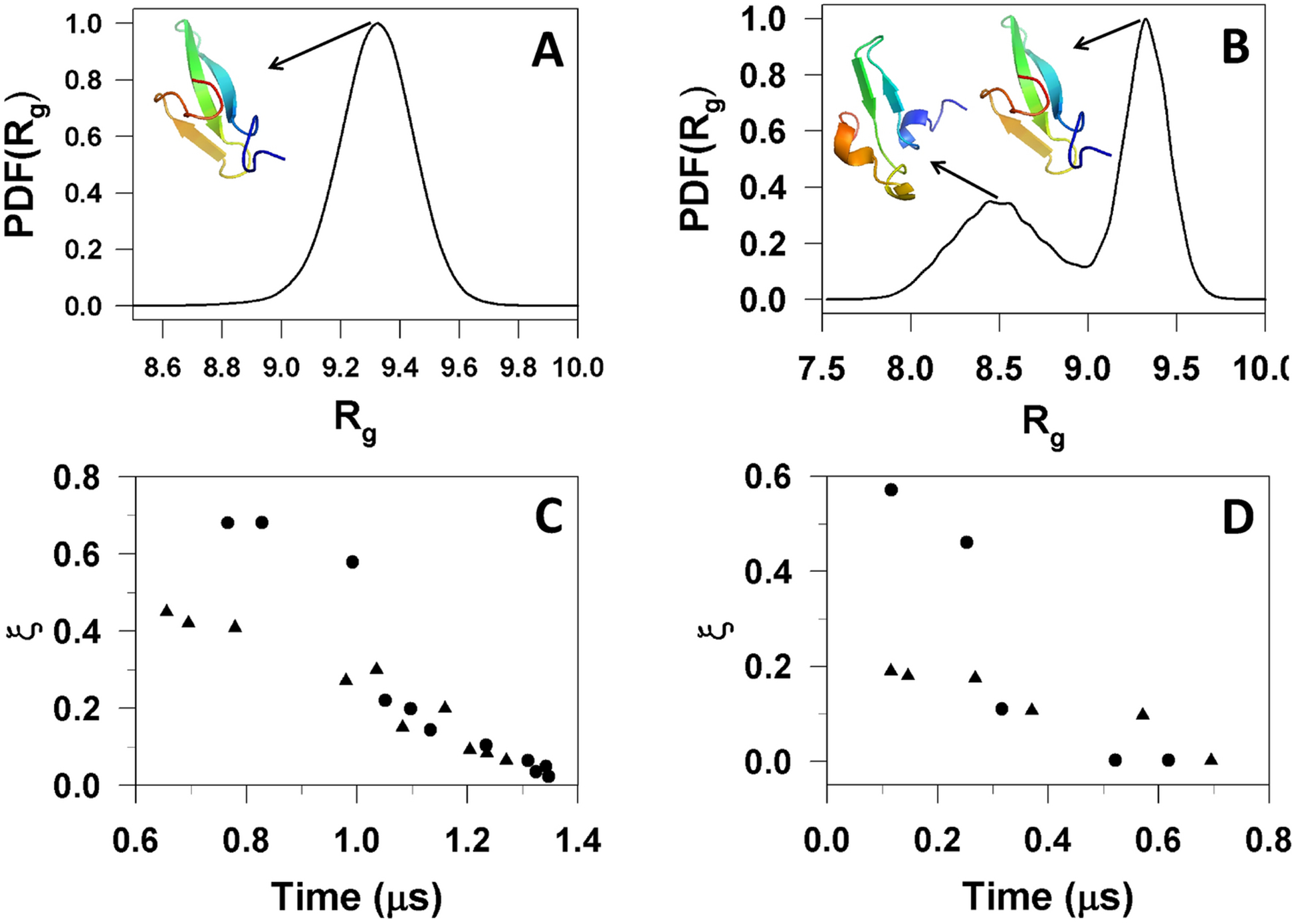
The probability distribution function of Rg for fast-folding (A) and slow-folding (B) trajectories of L26D with representative structures at the peaks. Dependence of the line-damping parameter ξ on time that protein remains in the native (C) and unfolded (D) states.The triangles and circles correspond to trajectories at 305K and 315K, respectively

**Fig. 4:**
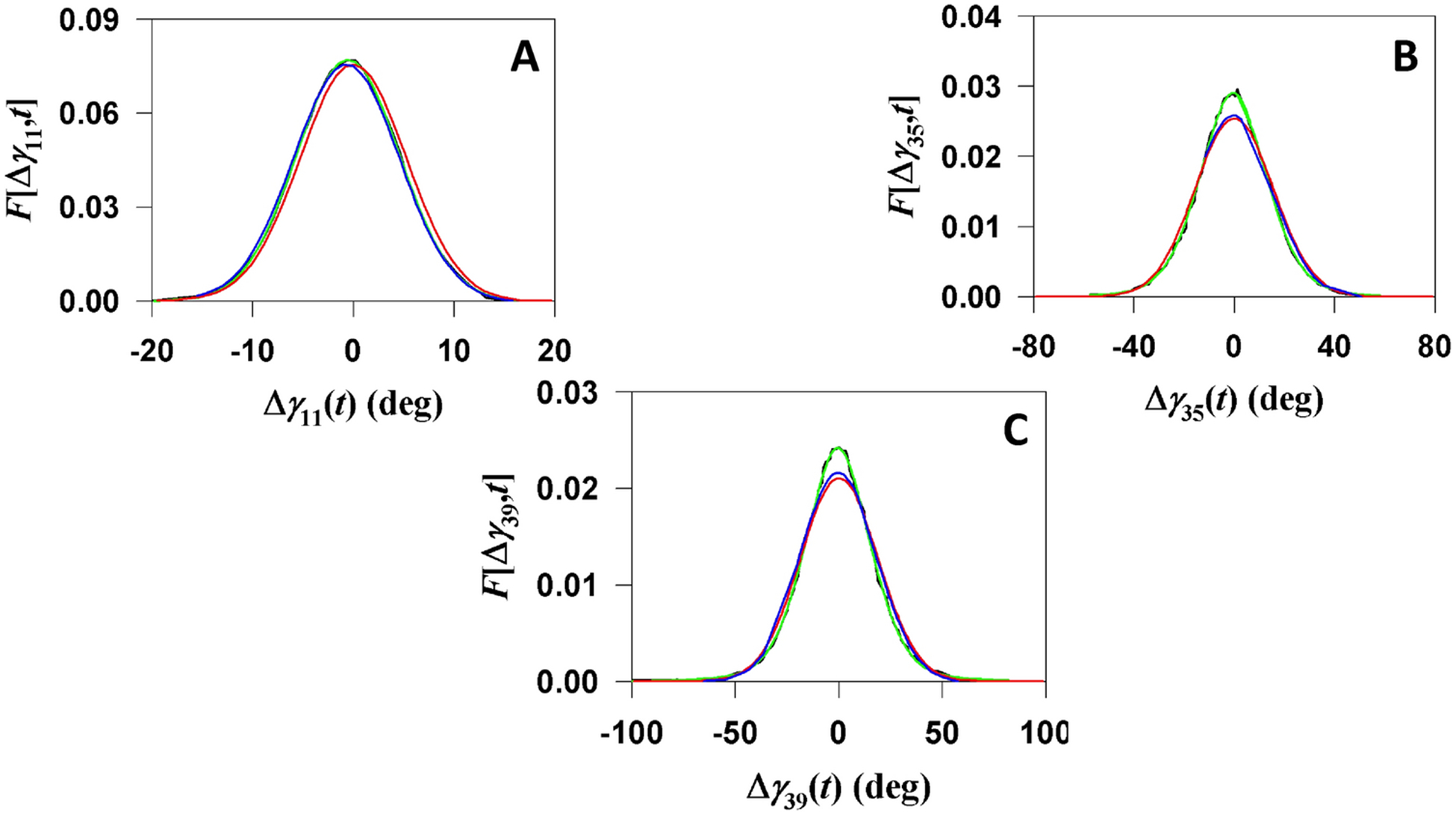
The probability distribution functions *F*(Δ*γ*_*n*_; *t*) for *γ*_11_ (A), *γ*_35_ (B), and *γ*_39_ (C) of VA3 computed for the MD trajectory (black lines), and evaluated by the Chebyshev polynomials (Eq. S17 in ref. [40]) (red lines), by the Gaussian function (Eq. S30 in ref. [40]) (blue lines), and by the Voigt profile (green lines)

**Fig. 5:**
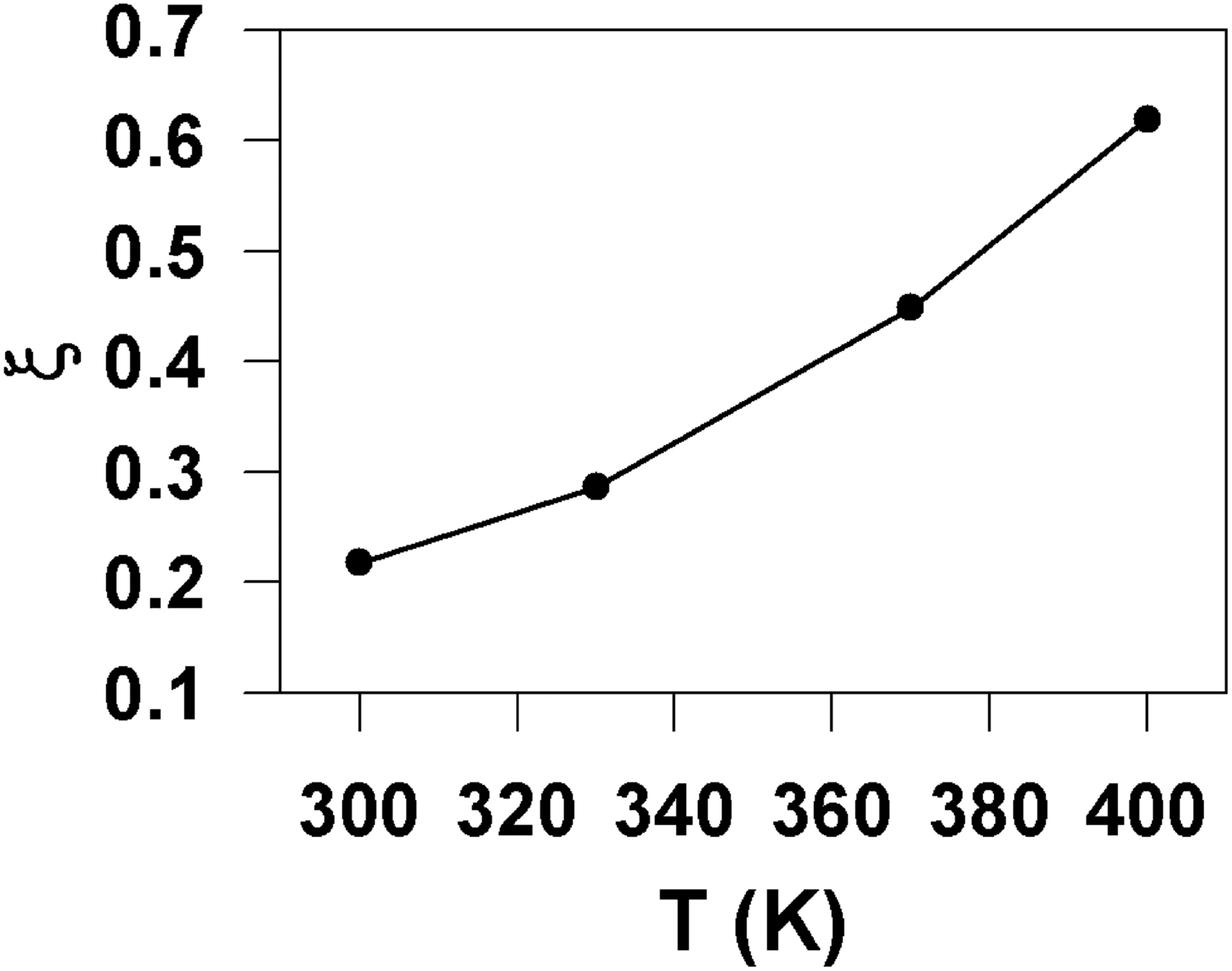
The line-damping parameter ξ, obtained from fitting quasielastic neutron-scattering spectra of lysozyme in deuterated glycerol, as a function of temperature
